# Inhibition of Rho-kinase by fasudil contributes to the modulation of the synaptic plasticity response in the rat hippocampus

**DOI:** 10.1007/s00424-025-03078-4

**Published:** 2025-04-12

**Authors:** Ercan Babur, Hatice Saray, Cem Süer, Nurcan Dursun

**Affiliations:** Department of Physiology, Erciyes University Faculty of Medicine, Kayseri, 38000 Turkey

**Keywords:** Hippocampus, Synaptic plasticity, ROCK, Fasudil, Protein Tau

## Abstract

Metaplasticity refers to an activity-dependent change in the physiological or biochemical state of neurons that changes their ability to generate subsequently induced synaptic plasticity, such as long-term potentiation (LTP) or long-term depression (LTD). Rho-kinases (ROCK) are known to be important for stable changes in synaptic strength, especially LTP. In this study, we investigated whether LTP inhibition in synapses primed with 1-Hz stimulation was affected by ROCK inhibition in young adult male rats. The study also examined the pattern of tau phosphorylation that occurs during metaplastic regulation, applying into perspective the phosphorylation of tau protein by ROCK. Field potentials consisting of an excitatory postsynaptic potential (fEPSP) and population spike (PS) were recorded from the granule cell layer of the hippocampal dentate gyrus (DG). Metaplastic LTP was induced by strong tetanic stimulation (HFS) of the lateral perforant path after a low-frequency stimulation (LFS) protocol. A glass micropipette was inserted into the granule cell layer of the ipsilateral dentate gyrus to record fEPSP and drug infusion. Drug infusion (saline, *n* = 8; fasudil, *n* = 8, 10 µM) was started after the 15-min baseline recording and lasted for 60 min. Total and phosphorylated tau levels were measured in the stimulated hippocampus, which was immediately removed after the electrophysiological recording. LFS prevented the induction of LTP in response to HFS and even produced synaptic LTD in the saline-infused group (83.8 ± 2.6% of the baseline), but moderate potentiation of fEPSP (121.1 ± 7.7% of the baseline) occurred at the end of recording in the experiments where fasudil infusion was performed. LFS caused a comparable early depression, and HFS resulted in a comparable potentiation of the PS amplitude in both groups. Granular cells of the DG failed to exhibit synaptic LTP inhibition in the presence of fasudil, and levels of total and phosphorylated GSK-3β and levels of phosphorylated tau (Ser^396^ and Ser^202^-Thr^205^) were found to be lower than those of the control group. Based on these findings, it can be concluded that pharmacological inhibition of ROCK results in impaired ability of dentate gyrus neurons to inhibit synaptic LTP, and this result is accompanied by decreased phosphorylation of GSK-3β and tau proteins. The negative effect of fasudil on neuronal function should not be neglected when evaluating its effects as a therapeutic agent for diseases.

## Introduction

Synaptic plasticity is a process in which synaptic strength changes in an activity-dependent manner within the brain. Experimentally, high-frequency stimulation (HFS) of presynaptic fibers can boost synaptic strength (long-term potentiation, LTP), whereas low-frequency stimulation suppresses it (long-term depression, LTD) [8]. Activation or inactivation of intracellular molecular cascades mediates these processes by altering the number and composition of ionotropic α-amino-3-hydroxy-5-methyl-4-isoxazolepropionic acid (AMPA)-type glutamate receptors (AMPARs) within the postsynaptic membrane. Activity-dependent modulation of AMPAR trafficking plays an important role in the expression of NMDA receptor-dependent LTP and LTD in the hippocampus [19]. LTP and LTD can be induced within seconds or minutes; however, their permanence depends on protein synthesis.

Tau phosphorylation may serve as a regulatory mechanism to avoid NMDA receptor over-excitation, demonstrating its vital role in regulating synaptic function. Interestingly, many kinases that play critical roles in synaptic plasticity are involved in abnormal tau phosphorylation [10, 23]. Under physiological conditions, a small fraction of tau in dendrites and spines is phosphorylated upon NMDA receptor activation through a signaling cascade that is also activated by the induction of synaptic plasticity [20]. We have previously shown that stimulation of the perforant pathway – dentate gyrus synapses by HFS increased the phosphorylation of total-tau and phospho-tau at Thr^181^, Ser^202^/Thr^205^, Ser^396^, and Ser^416^ residues in the hippocampus [29]. In contrast, LFS is unable to phosphorylate thr^181^ and thr^231^ epitopes of tau but possesses kinase activity similar to that of HFS in the phosphorylation of Ser^396^ and Ser^416^ epitopes [28]. As a result, we believe that synaptic plasticity-related tau phosphorylation may rise abnormally, potentially leading to neurodegenerative changes, in cases where one of the intracellular signaling pathways is primarily altered, even if plasticity remains within normal limits due to the compensatory effect of other intracellular pathways.

The Rho family of small GTPase RhoA and its downstream effector Rho-kinase (ROCK) are considered major regulators of synaptic plasticity and dendritic spine formation, including LTP. Pharmacological inhibition of ROCKs decreases tau phosphorylation in cellular models of tauopathy [9], and ROCK inhibitors represent a viable therapeutic route for reducing the pathogenic forms of tau protein in tauopathies [12]. We found that in the presence of the Rho kinase (ROCK) inhibitor, fasudil favors the induction of LTD over LTP. Furthermore, reduced HFS-LTP was accompanied by decreased Thr^181^ phosphorylation, while enhanced LFS-LTD was accompanied by decreased Thr^231^. In addition, ser^416^ phosphorylation was increased following LTP and LTD [26].

The mechanism by which ROCKs regulate synaptic plasticity is not fully understood. ROCKs, a member of the serine − threonine kinase family, can be activated by small GTPases, the inhibition of which affects the function of many downstream substrates [7]. Researchers have identified two mammalian forms of ROCKs, ROCK1 and ROCK2 [5]. Because of auto-inhibition, ROCKs are inactive in their native form [16]. Phosphatase and tensin homologs regulate the ROCK signaling pathway by altering the ability of phosphatidylinositol (PI)−3,4,5-triphosphate (PIP3) to produce Akt [30]. When the PI3K/Akt pathway is inhibited, the ROCK pathway is upregulated through Rho, leading to the activation of various enzymes, such as LIM-domain-containing protein kinases (LIMK) [18], myosin light chain kinase (MLC), and phosphatase [17]. Numerous studies have demonstrated that LIMKs are involved in both LTP and LTD. For instance, the LIMK1/cofilin pathway affects the trafficking and accumulation of AMPA at the synapse following LTP induction [11, 24]. However, after inducing LTP, activating the PI3K-Akt pathway inhibits GSK-3β activity, preventing synapses from expressing LTD for up to 1 h [23].

The ability to generate activity-dependent synaptic plasticity in a pool of neurons is dynamically regulated by the prior activity history [1]. Prior activity history established by priming stimulations at low frequencies (1–10 Hz) may influence the threshold for subsequent LTP, resulting in LTP inhibition. Although prior investigations have shown that ROCK inhibition promotes LTD expression, its influence on LTP inhibition and related tau phosphorylation remains to be studied.

## Materials and methods

### Experimental animals

Experiments were performed in vivo on adult male Wistar Albino rats aged 2–3 months. The guiding principles for the care of laboratory animals approved by the Erciyes University were applied to the care and use of animals. Experimental animals were obtained from the Erciyes University Experimental Research and Application Center (ERAC, Kayseri, Turkey). They were housed in rooms with a controlled environment (20 °C temperature and 60% humidity, lights on at 8:00 and off at 20:00). The rats were fed tap water as drinking water and Purina rodents were fed ad libitum. On the day of the experiment, the rats were anesthetized with urethane and transferred to the experimental room. Sixteen animals were used in this study. The rats were divided into two groups according to the intra-hippocampal infusion solution: saline (*n* = 8) and fasudil (*n* = 8) infusion groups.

### Stereotaxic surgery

The rats were anesthetized with urethane (1.2 g/kg) by intraperitoneal injection and then placed on a stereotaxic frame (David Kopf Instruments) and the bite bar was adjusted to match the dorsoventral coordinates at the bregma and lambda. A lengthwise incision was made on the head of each rat and all connective tissues were removed using 3% hydrogen peroxide to expose the bregma. Using a hand drill, the skull in the right hemisphere was removed to expose the brain. Following an 8-mm craniotomy, bipolar stainless steel wire electrodes (127 µm in diameter, insulated except ends) were lowered into the angular bundle of the perforant pathway (PP) according to flat skull stereotaxic coordinates (6.5 mm posterior to bregma, 3.8 mm lateral to the midline). The stimulating electrode was connected to the output of a stimulus isolator (World Precision Instruments, USA). Next, a recording electrode was pulled from borosilicate 2-barrel glass capillaries with an external diameter of 1.5 mm (World Precision Instruments, outer diameter 1.5 mm, length 10 cm) through a vertical micropipette puller (P30, Sutter Instrument Co., USA). One barrel was filled with 3 M NaCl (tip resistance: 2–10 MΩ). An Ag/AgCl wire that was in contact with this solution and a reference electrode (An Ag–AgCl disc electrode positioned under the neck skin) were connected to an amplifier (MultiClamp 700B Axon CNS, Computer Controlled Current and Voltage Clamp, Molecular Devices, USA) using a head-stage. The other barrel was filled with the drug (NaCl or fasudil) to be infused and connected to a Hamilton syringe (25 µL) driven by a syringe pump (Stoelting Co., Wood Dale, IL, USA) via polyethylene tubing. The recording electrode was then lowered into the dentate gyrus (DG; bregma, in mm: AP: − 3.5, ML: 2.15). The recording and reference electrodes were connected to the entire system and shielded using Faraday cages. The coordinates used for both electrodes were based on the atlas of Paxinos and Watson [22] and were previously confirmed to be in the granule cell layer of the DG and PP [27]. The effect of anesthesia lasted for 2–3 h and no additional anesthetic dose was required during electrophysiology.

#### Recording of field potentials

We recorded the field potentials composed of a positive excitatory postsynaptic potential (fEPSP) and a negative population spike (PS) from the DG, digitized at a sampling rate of 40 kHz, and stored them on a computer for later analyses using SCOPE software (PowerLab ADInstruments, version 3.6.10). The depths of the recording and stimulating electrodes (dorsoventral coordinate) were adjusted to achieve a large positive fEPSP, followed by a negative PS.

### Obtaining the input–output (I/O) curves

The I/O curves were obtained by plotting the raw values of the fEPSP slope and PS amplitude against an electrical pulse varying from 0.1 to 1.5 mA. For this, the PP was stimulated at a frequency of 0.05 Hz with 0.2 mA steps after 15 min of stable baseline recordings. A pulse intensity that produced half of the maximum PS amplitude (test stimulus) was used throughout the experiment.

### Induction of metaplastic long-term potentiation

Following the I/O curve protocol, electrical stimulation of the PP continued for 110 min at a frequency of 0.33 Hz (one pulse every 30 s). Drug infusion (saline or fasudil) was started after the 15-min baseline recording and lasted for 60 min. A Hamilton pump infused a 20 µL solution into the DG at a rate of 0.33 µL/min. Simultaneously with the start of the infusion, LTP was induced by applying strong high-frequency stimulation (HFS, 100 Hz, 1-s duration, 5 min interval, 4 times) following priming stimulation with low-frequency stimulation (LFS, 1 Hz).

### Hippocampectomy

Immediately after completion of electrophysiology, in a deeply anesthetized rat, the brain was carefully dissected out from the skull, placed into ice-cold phosphate-buffered saline, cut along the longitudinal fissure of the cerebrum using a surgical knife, and cut off the regions posterior to the lambda (midbrain, hindbrain, and cerebellum). The medial side of the cerebral hemisphere was then placed and the diencephalon (thalamus and hypothalamus) was carefully removed under a dissection microscope, allowing visualization of the hippocampus. Using forceps, the right and left hippocampi were dissected separately from the surrounding area and weighed.

### Chemicals

Fasudil (Lot: FCB059815) was purchased from Flourochem Company and dissolved in DMSO in a minimal volume of 10 mM. The prepared chemical agents were diluted in saline. Saturated stock solutions 1 M were prepared using the diluted drug. The drug form was then diluted to 10 µM (final concentration) and infused into the fasudil group.

The primary antibodies used in western blot analysis were as follows: mouse mAb-Tau (Tau46; 1:1000, 4019, Cell Signaling), and rabbit mAb-p-Ser416 -Tau (1:1000, D7U2P, 15,013, Cell Signal). Ab-p-Ser416-Tau (1:1000, D7U2P, 15,013, Cell Signaling), p Ab-p-Ser 396-Tau (1:1000, 44-752G, Invitrogen, Thermo Scientific), m Ab-p-Thr181-Tau (1:500, AT270, MN1050, Invitrogen, Thermo Scientific), Ab-β-Thr231-Tau (1:1000, PHF-6, sc-32276, Santa Cruz Biotechnology), and Ab-β-actin (1:1000, AC-15, sc-69879, Santa Cruz Biotechnology). The secondary antibodies, mouse anti-rabbit IgG-HRP (sc-2357, Santa Cruz Biotechnology) and anti-mouse m-IgGk BP-HRP (sc-516102, Santa Cruz Biotechnology), were used.

### Determination of protein levels by western blot

The entire hippocampus was dissected from the brain immediately after recording and lysed in RIPA buffer (sc24948; Santa Cruz Biotechnology, Santa Cruz, California, USA) with thorough homogenization. This buffer was supplemented with a protease inhibitor cocktail, PMSF, and sodium orthovanadate (sc-24948; Santa Cruz Biotechnology), and 10 µL of each was added to 1 mL of RIPA buffer on ice immediately before use. Homogenates were centrifuged at 15,000 × g for 20 min at 4 °C. Forty micrograms of total protein from each sample was separated on a 10% SDS polyacrylamide gel and transferred onto a PVDF membrane (GE Healthcare 10,600,021 Amersham Hybond, UK). The membranes were blocked with 5% non-fat dry milk in phosphate-buffered saline (PBS) containing 0.1% Tween20 (PBS-T) for 1 h at room temperature before being incubated with the primary antibodies overnight at 4 °C. The membranes were then washed with TBST and incubated for 1 h at room temperature with a 1:4000 dilution of goat anti-rabbit IgG (sc2004; Santa Cruz Biotechnology) or m-IgGκ BP-HRP (sc-516102, Santa Cruz Biotechnology) secondary antibodies conjugated to horse radish peroxidase. The bound antibodies were visualized by a Syngene G:Box XR5System (Beacon House, Cambridge, UK) using an enhanced chemiluminescence substrate (Pierce™ ECL Western Blotting Substrate, Catalog number: 32106) according to the manufacturer’s instructions.

The blots were subsequently stripped and re-probed with a β-actin mouse monoclonal antibody (AC-15, sc-69879– Santa Cruz Biotechnology) to confirm equal loading of protein samples in the gel (limited to a maximum of two loads). The optical density (OD) of the blots was measured using the Image J software (National Institutes of Health, USA). The OD of each band was normalized to that of the corresponding β-actin band. The relative OD (ROD) was calculated by dividing the optical density of the analyzed sample by the first band of each blot.

### Data analysis and statistics

The slope of the EPSP was calculated as the amplitude change at 20–80% of the voltage difference between the start and peak of the waveform. The PS amplitude was calculated as the average of the two potential differences between the negative spike peak and the preceding and following positive peaks. The mean value of the EPSP slope and PS amplitude during the baseline recording was chosen to represent 100%, and each EPSP slope and PS amplitude were expressed as a percentage of this value. The ratio of the 5-min averages of the EPSP slopes and PS amplitudes at the end of the recording was used as a measure of the magnitude of LTP.

The EPSP slope and PS amplitude in the I/O curves were analyzed for significance using repeated-measures analysis of variance (ANOVA) with stimulus intensity (eight levels of intensity) as a within-subjects factor. LTP magnitude was analyzed for significance using the Student’s *t*-test. Statistical analysis of the western blots was performed using the Mann–Whitney *U* test. A post-hoc independent *t*-test was performed, when appropriate. Statistical significance was set at *p* < 0.05 (two-tailed). SPSS software (version 15.) was used for the statistical analysis.

## Results

Before metaplasticity induction, understanding that the group to be infused with fasudil does not differ from the group to be infused with saline in terms of baseline synaptic strength and somatic output is important for interpreting plasticity differences. For this, the I/O curves were obtained by increasing stimulus intensity from 0.1 to 1.5 mA at 0.2 mA steps before infusions, showing that both groups exhibited similar baseline synaptic strength and neuronal output. As expected, the EPSP slope and PS amplitude increased systematically as a function of stimulation intensity as a result of stimulation of the perforating pathway (repeated measures ANOVA, *p* < 0.001). There was no significant interaction between the group and stimulus intensity (*p* > 0.05, data not shown).

Many researchers have observed LTP-like increases in the EPSP slope and population spike amplitude of perforant path-dentate gyrus evoked potentials as a result of electrical stimulation compared to those evoked before stimulation. Although increases in the fEPSP slope have been evaluated as an indicator of synaptic plasticity in most studies, increases in the PS amplitude can also be observed parallel to or independent of this increase.

### The LTP of fepsp slope

**Fig. 1:**
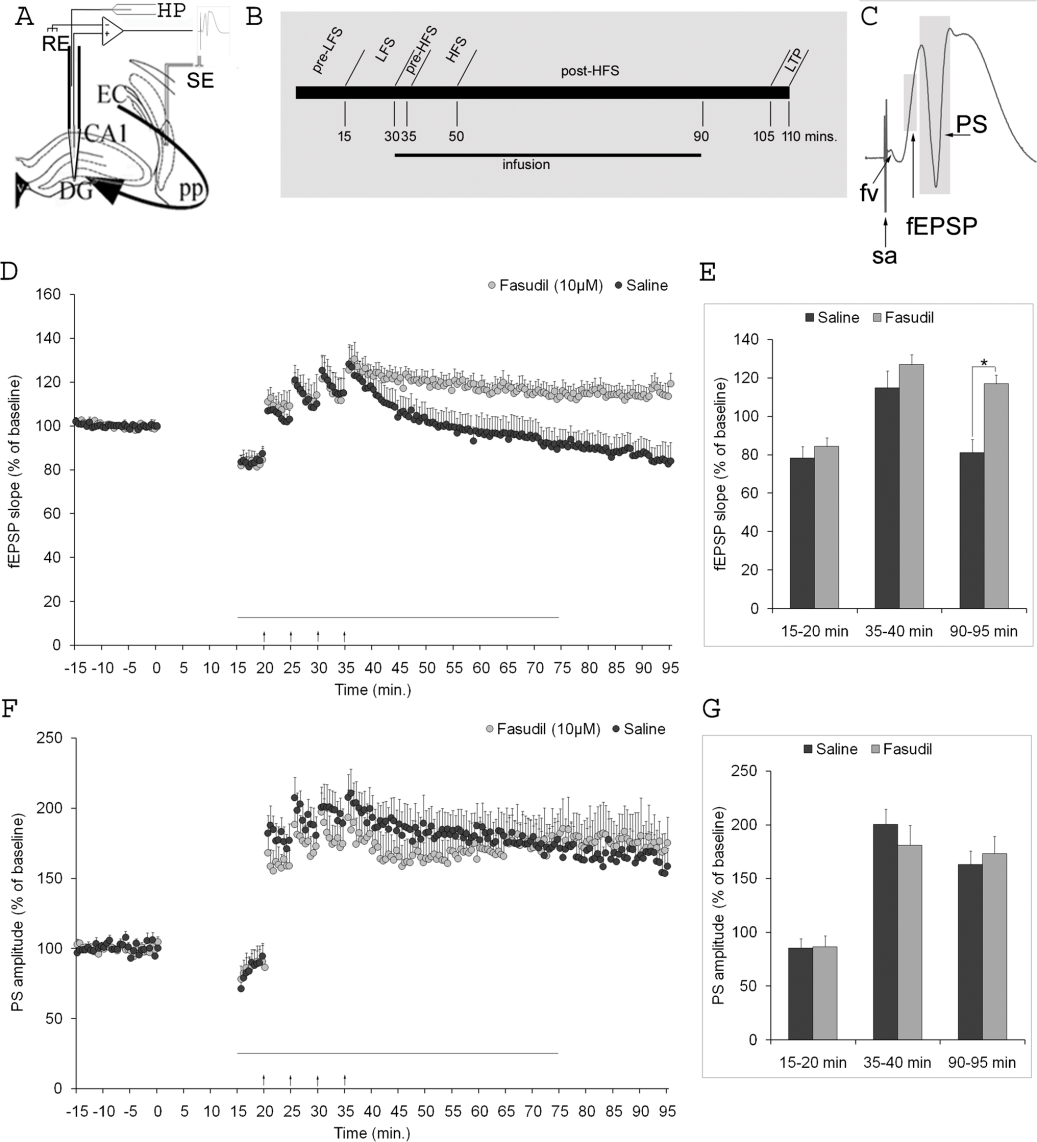
Inhibition of ROCK with fasudil can reverse inhibition of LTP induced after LFS. **A** Experimental setup and schematic representation of electrode placement. Recording electrode (RE) inserting into the dentate gyrus (DG) and a reference electrode were connected to an amplifier. The stimulating electrode (SE) was placed onto the perforant pathway (pp). Infusion was made into the DG using a Hamilton pump (HP). EC, entorhinal cortex; CA1, cornu ammonis region of the hippocampus proper. **B** Schematic representation of recording protocol. LFS, low-frequency stimulation; HFS, high-frequency stimulation, LTP, long-term potentiation. **C** A sample trace of hippocampal field potential composing of field excitatory postsynaptic potential (fEPSP) and population spike (PS) components. fEPSP slope was calculated as the change between 20 and 80% of the first upward deflection per unit time. The PS amplitude was calculated as the average of the two potential differences of the negative spike peak to the preceding and following positive peaks. The excitatory postsynaptic potential (EPSP) slope **(D**) and population spike (PS) amplitude (**F**) in the dentate gyrus of hippocampal formation. Long-term potentiation was induced with 1-s tetanic pulses at a frequency of 100 Hz (arrows), 5 min after electrical stimulation at a frequency of 1 Hz (between 0 and 15 min), lasting 15 min under saline (*n* = 8, filled circle, one data was excluded from the fEPSP analysis) and fasudil (*n* = 8, grey circle, one data was excluded from the fEPSP analysis) infusion (horizontal bar). **E** The mean fEPSP slopes and statistical analysis were calculated at 5-min time intervals. *p < 0.001; Student *t*-test. **G** The mean PS amplitude and statistical analysis were calculated at 5-min time intervals

Both the studies of our group and numerous studies in the literature have shown that tetanic stimulation of the perforant pathway can induce long-term synaptic plasticity in the dentate gyrus. In this study, we found that LFS inhibited HFS-induced synaptic LTP and even produced synaptic LTD in the saline-infused group (Fig. 1D). In this group, the fEPSP slope, which decreased by 83.8 ± 2.6% of its basal value after LFS, was potentiated by 121.1 ± 7.7% with HFS administration but decreased by 84.6 ± 7.1% at the end of 1 h recording. In the experiments where fasudil infusion was performed, although similar values were measured for post-LFS depression (83.1 ± 4.9%) and post-HFS potentiation (125.7 ± 5.9%), the EPSP slope after 1 h was potentiated (115.5 ± 4.8%). A statistically significant difference was found between the mean fEPSP slopes in the last 5 min of the recording (*t*_12_ = 4.450; *p* = 0.003; Fig. 1E). These results demonstrate that fasudil can prevent metaplastic LTP inhibition.

### The LTP of PS amplitude

LFS caused a comparable early depression of PS amplitude in the saline group (85.5 ± 8.6%) and fasudil infusion group (86.7 ± 9.9%). The PS amplitude showed similar increases with HFS given subsequently, reaching an increase of 162.9 ± 12.8% in the saline infusion group and an increase of 173.1 ± 16.3% in the fasudil infusion group in the last 5 min at the end of the experiment (Fig. 1F). These findings indicate that LFS was unable to inhibit subsequent PS-LTP induction and that metaplastic somatic LTP was not altered by fasudil infusion (Fig. 1G).

**Fig. 2:**
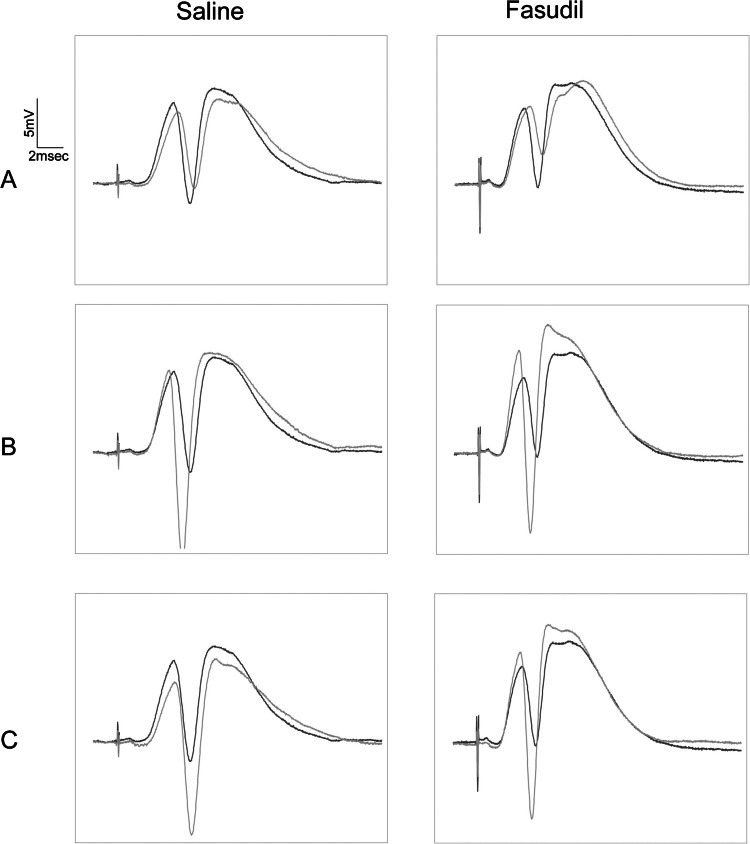
Field potential traces reveal that fasudil affects LFS results in the prevention of HFS-induced LTP in DG granule cells. The black traces represent the field potential right before LFS application, while the gray traces represent the field potential just after LFS (**A**), shortly after HFS (**B**), and 60 min after HFS (**C**). In the experiment with saline infusion, the slope of the EPSP component showing the change in synaptic strength decreases immediately after LFS (left panel, **A**), increases immediately after HFS (left panel, **B**), and decreases dramatically 60 min later (left panel, **C**). In contrast, the EPSP slope increased in the experiment performed with fasudil infusion (right panel, **C**)

In light of these findings, it can be concluded that pharmacological inhibition of ROCK results in impaired ability of dentate gyrus neurons to inhibit synaptic, but not somatic LTP. Figure 2 depicts the field potentials derived from the two rats, which reflects this electrophysiological conclusion.

### Molecular studies

**Fig. 3:**
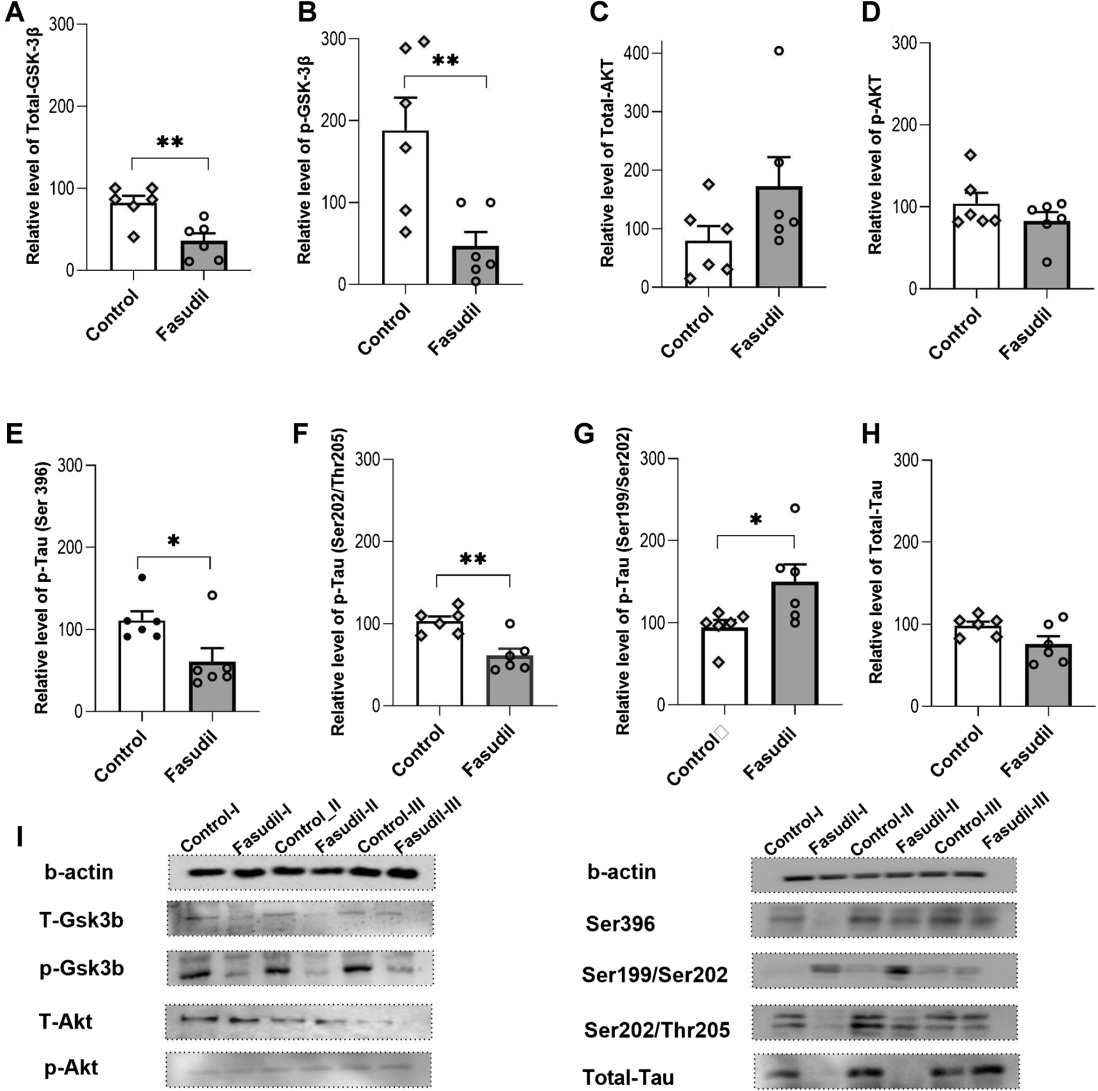
The effects of fasudil infusion on metaplasticity-induced hippocampal tissue. Protein levels Total-GSK-3β (**A**), p-GSK-3β (**B**), Total-AKT (**C**), p-AKT (**D**), p-Tau (Ser396) (**E**), p-tau (Ser202/Thr205) (**F**), and p-Tau (Ser202/Thr205) (**G**) were measured by Western blot in the 12-week-old rat hippocampus (*n* = 6). Values represent the mean ± SEM. The significant level between the groups is indicated by *p < 0.05 and **p < 0.01

The levels of Tau, AKT, and glycogen synthase kinase 3 (GSK-3β) protein were determined by western blotting in hippocampal tissues removed at least 70 min after metaplastic LTP induction (Fig. 3). Lower total GSK-3β (Fig. 3A; *t*_10_ = 3.605; *p* = 0.005) and decreased ser9 phosphorylation of GSK-3β (Fig. 3B; *t*_10_ = 3.235; *p* = 0.009) levels were found in hippocampal tissues that failed to exhibit synaptic LTP inhibition in the presence of fasudil compared to the control group, which exhibited synaptic LTP inhibition. There was no significant difference in the total and phosphorylated levels of AKT between the groups (Fig. 3C and D; *p* > 0.05). In addition, phosphorylation of tau at Ser^396^ (*t*_10_ = 2.545; *p* = 0.029, Fig. 3E) and Ser^202^-thr^205^epitopes (*t*_10_ = 3.997; *p* = 0.003, Fig. 3F) was decreased in the fasudil-infused group compared to the control group, and phosphorylation at Ser^199^-Ser^202^ (*t*_10_ = 2.431; *p* = 0.0354, Fig. 3G) was increased. Although total tau levels decreased in the fasudil-infused group, this decrease was not statistically significant (*t*_10_ = 2.047; *p* = 0.006, Fig. 3H). Figure 3I shows band images illustrating alterations in protein levels within the groups.

**Fig. 4:**
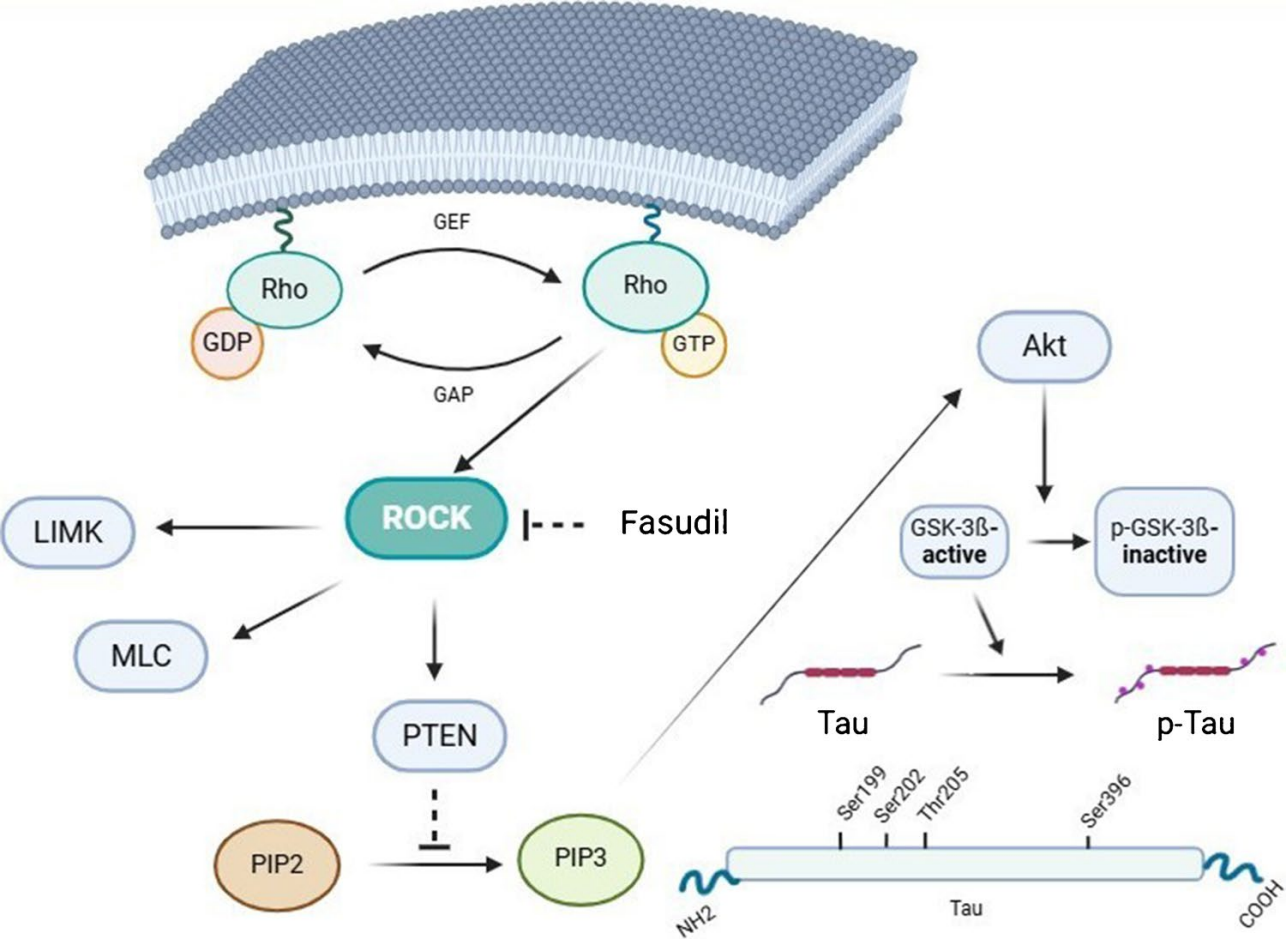
RhoA/ROCK signaling pathway and Tau. Rock reduces Akt activity through the activation of PTEN, an inhibitory protein. Decreased Akt activity reduces GSK-3β phosphorylation and thus its inhibition. As a result, increased GSK-3β activity results in tau hyperphosphorylation. Abbreviations: GEF, Guanine Nucleotide Exchange Factor; GAP, GTPase-activating protein; GSK-3β, glycogen synthase kinase-3β; LIMK, LIM kinase; MLC, myosin light chain; PIP2, phosphatidylinositol 4,5-bisphosphate; PIP3, phosphatidylinositol (3,4,5)-trisphosphate; ROCK, Rho-associated coiled-coil protein kinase

## Discussion/Conclusion

The present in vivo experiments demonstrated that LFS-induced LTD obstructed the ability of young adult male rats to express HFS-induced LTP in the dentate gyrus. This metaplastic inhibition of LTP was previously demonstrated in the cornu ammonis 1 (CA1) region of hippocampus slices [14]. Metaplasticity appears to be critical for maintaining synaptic memory traces and synaptic plasticity within a dynamic range [21]. The specific mechanisms of metaplasticity remain unclear; however, a decrease in successive LTP appears to be based on NMDA activity and lasts from to 30–90 min [13]. An attractive explanation for NMDAR-dependent metaplasticity is the long-term alteration of enzymes that regulate synaptic plasticity by previous synaptic “priming” [3]. We previously reported that both HFS-induced neuronal plasticity and LFS-induced neuronal plasticity were attenuated by fasudil, an inhibitor of Rho-associated kinases [26]. The present study describes the ability of the DG to produce LTP at synapses that have been primed with low-frequency activity and demonstrates that metaplastic inhibition of LTP is defective under pharmacological inhibition of ROCK by fasudil. Considered together with our previous study, the present findings support the important role of the Rho/ROCK signaling network in the regulation of synaptic plasticity (Fig. 4). Inhibition of the Rho/ROCK signaling pathway has also been shown to improve LTP deficiencies associated with certain neuropathologies. In an experimental study using a brain ischemia model, long-term inhibition of ROCK improved the impaired LTP by regulating gamma-aminobutyric acid A (GABA A) and GABA B receptors in the brain tissue [5]. The neuroprotective effects of fasudil may also be due to decreased cerebral blood flow, inflammatory responses, tau phosphorylation, actin cytoskeleton reorganization, and apoptosis. In addition to these neuroprotective effects, our study indicates an inhibitory effect of locally applied fasudil on the metaplastic control of neuronal activity.

In this study, the findings obtained from the control group showed that LFS inhibits synaptic LTP but cannot prevent the potentiation of neuronal output. The LTP of the EPSP provides direct information about synaptic plasticity, as it depends on presynaptic inputs. However, LTP of the PS represents an increase in the number of neurons that fire simultaneously, depending on alterations in intrinsic excitability and synaptic strength. Therefore, it can be argued that the LTP suppressing effect of LFS occurs mostly at the synaptic level, independent of changes in neuronal excitability. In contrast, fasudil appears to prevent the inhibitory effect of LFS at the synapse level but does not affect neuronal excitability. The increasing effect of fasudil on synaptic protein expression appears to be consistent with our findings. Fasudil enhances the expression of PSD-95 and Syn in oxygen-glucose-deprived SH-SY5Y cells [32]. However, fasudil may also affect synaptic transmission via a presynaptic mechanism. A study of primary rat hippocampal cultures found that fasudil does not alter postsynaptic structure but accelerates vesicle turnover and decreases the number of released vesicles [25]. In contrast, regulation of neuronal excitability is mainly achieved by voltage-gated K channels. Although the vasorelaxant effect of fasudil is attributed to the selective activation of vascular Kv7.4/Kv7.5 K^+^ channels [31], the present study does not support the long-term effect of fasudil on K^+^ channels.

Priming the presynaptic input immediately before HFS administration alters the form of plasticity to expressed in postsynaptic neurons. The current study showed that LTP, which is expected to be expressed after HFS, is inhibited by LFS. Moreover, fasudil prevented metaplastic inhibition of synaptic LTP. It is difficult to assess whether there is a relationship between the metaplastic effects and fasudil without data on the effect of fasudil on LTP. Our previous research indicated that fasudil reduced HFS-induced neuronal plasticity while leaving the baseline synaptic strength unaffected [26], whereas the current study demonstrated that the inhibitory effect of low-frequency stimulation on long-term potentiation caused by high-frequency stimulation was prevented by fasudil administration. These findings not only demonstrate the contribution of Rho-kinases to synaptic plasticity but also support the dependence of the effect on the induction method.

GSK-3β is an unusual enzyme because it has high basal activity, which is primarily determined by the phosphorylation status of the Ser^9^ epitope. The dephosphorylation of this residue by Ser/Thr protein phosphatases leads to further activation of GSK3β, whereas phosphorylation of ser^9^ by a variety of kinases results in inhibition of its activity. Our molecular findings indicated that fasudil-induced disinhibition of metaplastic LTP is accompanied by a decrease in the inhibitory phosphorylation of GSK-3β. As GSK-3β is an effective tau kinase, this effect is expected to be accompanied by an increased tau phosphorylation pattern. Indeed, fasudil significantly reduced phosphorylation of the Ser^396^ and Ser^199^-Thr^202^ residues. Fasudil, however, significantly reduced phosphorylation of the Ser^396^ and Ser^202^-Thr^205^ residues. In contrast, in our previous study, fasudil down-regulated Thr^181^-Tau expression levels after HFS and Thr^231^-Tau expression levels after LFS [26]. Although these findings indicate the importance of tau phosphorylation in synaptic regulation, more detailed studies are required to understand the relationship between metaplastic LTP inhibition and tau phosphorylation.

In neurodegenerative diseases, tau aggregation is associated with post-translational modifications. An increase in tau phosphorylation may affect the hydrophilicity, spatial conformation, and stability of the tau protein, promoting tau protein aggregation and the formation of neurofibrillary tangles. These tangles are characterized by high phosphorylation of tau proteins and are involved in the regulation of microtubule dynamics. Some of the hypotheses regarding abnormal phosphorylation of Tau proteins include phosphorylation of Tau by GSK-3β or cyclin-dependent kinase 5 (Cdk5), and other Tau-related kinases [18]. In previous studies, selective inhibition of ROCKs has been shown to alleviate the pathologies caused by tauopathies [17]. ROCKs are considered therapeutic targets for several neurodegenerative disorders, including stroke, Alzheimer’s, disease [4], Parkinson’s disease (PD) [2], and Amyotrophic Lateral Sclerosis (ALS) [6, 15]. Although the findings of this study were obtained from healthy young adult rats, reduced tau phosphorylation accompanying metaplastic regulation suggests a protective effect of fasudil-induced ROCK inhibition.

As a result, this study is the first to show that ROCKs are involved in metaplastic LTP regulation. Based on our findings, we speculate that fasudil disrupts processes that prevent LTP from reaching saturation levels by altering tau phosphorylation. These findings highlight the need for detailed studies to evaluate ROCKs as therapeutic targets for neurodegenerative diseases.

## Data Availability

No datasets were generated or analysed during the current study.
